# Pain and daily interference among reproductive-age women with myofascial pelvic pain: Serial mediation roles of kinesiophobia, self-efficacy and pain catastrophizing

**DOI:** 10.1371/journal.pone.0301095

**Published:** 2024-05-13

**Authors:** Mingyu Si, Juan Chen, Xue Zhang, Lan Zhu, Yu Jiang

**Affiliations:** 1 School of Population Medicine and Public Health, Chinese Academy of Medical Sciences & Peking Union Medical College, Beijing, China; 2 Department of Obstetrics and Gynecology, National Clinical Research Center for Obstetric & Gynecologic Diseases, Peking Union Medical College Hospital, Chinese Academy of Medical Sciences & Peking Union Medical College, Beijing, China; 3 School of Health Policy and Management, Chinese Academy of Medical Sciences & Peking Union Medical College, Beijing, China; Ningbo University, CHINA

## Abstract

**Background:**

Myofascial pelvic pain (MFPP), which is identified by tender points in the pelvic floor musculature, is a prevalent source of chronic pelvic pain in women. It may lead to physical and mental exhaustion, reproductive concerns, and coping difficulties in daily life and work than the disease itself. Pain-related cognitive processes can affect pain relief and quality of life. Kinesiophobia, self-efficacy and pain catastrophizing are frequently treated as mediators between pain and its related consequences. Greater kinesiophobia and pain catastrophizing have been shown to be associated with adverse functional outcomes, while higher self-efficacy has been related with improved quality of life. Regarding MFPP in females of childbearing age, it remains unclear whether the effects of kinesiophobia, self-efficacy and pain catastrophizing on daily interference are direct or indirect; the influence on each variable is, therefore, not entirely evident.

**Aim:**

The present study aimed to evaluate the relationship between pain and daily interference in reproductive-age women with MFPP through kinesiophobia, self-efficacy and pain catastrophizing, as well as to identify areas for future investigation and intervention based on the data collected from this population.

**Methods:**

This is a multi-center cross-sectional study. The study was conducted from November 15, 2022 to November 10, 2023, 202 reproductive-age women with MFPP were recruited from 14 hospitals in ten provinces of China. The demographic variables, Brief Pain Inventory, Tampa Scale of Kinesiophobia, Pain Self-Efficacy Questionnaire, and Pain Catastrophizing Scale were used to measure the participants’ related information. The data was described and analyzed using Descriptive analyses, Pearson correlation analysis, and Serial mediation modeling.

**Results:**

Pain not only had a direct positive impact (*B* = 0.575; *SE  * =   0.081; *95%CI*: LL = 0.415, UL = 0.735) on daily interference, but also had an indirect impact on daily interference through the independent mediating role of pain catastrophizing (*B* = 0.088; *SE * =  0.028; *95%CI*: LL = 0.038, UL = 0.148), the chain mediating of kinesiophobia and catastrophizing (*B* = 0.057; *SE * =   0.019; *95%CI*: LL = 0.024, UL = 0.098), and the four-stage serial mediating of kinesiophobia, self-efficacy and catastrophizing (*B* = 0.013; *SE  * =  0.006; *95%CI*: LL = 0.003, UL = 0.027). The proposed serial mediation model showed a good fit with the collected data.

**Conclusion:**

The findings illustrate the significance of addressing pain catastrophizing and kinesiophobia (especially catastrophizing), and increasing self-efficacy in pain therapy, and suggest that functional recovery be integrated into pain therapy for reproductive-age women suffering from MFPP.

## Introduction

Myofascial pelvic pain (MFPP) is distinguished by hypersensitive points in the muscle tissues around the pelvic floor that cause referred pain once triggered (i.e., stretching, unpleasant procedures, or emotional disturbances) [1]. Globally, the prevalence of MFPP in women with chronic pelvic pain is thought to range from 22% to 94%, and up to 85% of women with gynecologic, urologic, or colorectal pain issues also have MFPP [2, 3]. Latent trigger points can be asymptomatic and cause discomfort. However, as the pain worsens, it frequently interferes with many aspects of daily life, causing painful spasms, dyspareunia, emotional disorders, and sleep disturbance, as well as having negative effects on social life, work capability, and leads to high demands on sick leave [1, 4]. Patients of reproductive age (18–49 years old) are without a doubt the population that warrants significant attention due to their high prevalence of chronic pelvic pain [5]. Additionally, sexual pain or dyspareunia caused by MFPP makes their conception challenging. Moreover, patients are counseled against becoming pregnant until their pain relief or else run the danger of discomfort worsening as pregnancy progresses.

Pain intensity is the major determinant defining the impact of pain on the patient and the aim for therapy [4]. Furthermore, the fear-avoidance model (FAM) [6], a well-established biopsychosocial framework for clinical pain conditions, proposes that pain catastrophizing and fear of movement/(re)injury (i.e., kinesiophobia) are significant determinants of pain intensity and disability. The importance of self-efficacy in pain management has also been established in various kinds of chronic pain [7]. However, while it is generally recognized that there is a connection between pain intensity, the principal components of the FAM, self-efficacy and disruptions in daily life, the mechanisms that contribute to this relationship among reproductive-age patients with MFPP remain unclear.

The duration of pain and its interference following the onset of pelvic pain may be attributed to various causes, such as widespread pain, sensory incongruence, social disengagement, and a negative state of mind. The FAM, in particular, has been widely accepted to explain that it is not pain itself, but rather how pain is interpreted that is responsible for either speedy recovery or the progression of chronic pain. To begin with, it has been established that pain catastrophizing, a psychological condition characterized by an excessive focus on excruciating sensations and a feeling of helplessness while dealing with pain, is a significant and constant psychological predictor of reduced functioning [8]. For example, in 184 patients with chronic pelvic pain, Chen A et al. discovered a significant correlation between catastrophizing and severity and number of pain comorbidities [9]. Moreover, a review of 63 articles found evidence for an association between a greater degree of kinesiophobia and higher levels of pain severity and low quality of life [10]. MFPP, in particular, would be a consequence of central sensitization [11], which may interact with pain catastrophizing and kinesiophobia to trigger an exaggerated reaction towards pain, causing patients to compromise their function and, as a result, experience higher levels of daily interference [6].

While there is constant links between pain catastrophizing, kinesiophobia and worse outcomes, self-efficacy—that is, the belief in one’s capacity to carry out particular tasks—also plays a significant role in how one experiences and manages pain [12]. According to studies, individuals with similar pain levels typically experience less negative daily interference if they have a higher pain self-efficacy [7, 13]. Furthermore, a meta-analysis of 27 articles found promising findings regarding the impact of self-efficacy in the prognosis of chronic musculoskeletal pain [13], self-efficacy seems to facilitate pain recovery and lessen the negative effects of pain on routine activities (e.g., reduced pain severity, decreased functional impairment, enhanced quality of life). It is commonly considered in the existing literature that self-efficacy usually acts as a mediator between pain and pain-related outcomes [13]. However, evidence also suggests that one’s sense of efficacy is an independent psychological capacity that may serve as a protective factor against the development of undesirable events caused by one’s condition, such as pain-related outcomes, illness sequela, and poor quality of life [14]. In this regard, we seek to investigate if, in reproductive-age patients with MFPP, self-efficacy alone or together with pain catastrophizing and kinetophobia forms a chain pathway that mediates pain and daily interference.

To the best of our knowledge, only two studies—neither of which focused on reproductive-age women or the pelvic region—have found a relationship between pain, psychological characteristics (e.g., pain catastrophizing and self-efficacy), and daily interference among patients with chronic myofascial pain [15, 16]. According to Healy GM et al., patients with chronic myofascial pain who experienced a positive response to trigger point injection therapy had less anxiety before treatment and more self-efficacy in dealing with pain [15]. Similarly, Lumley MA et al. found that patients with chronic myofascial pain had less self-efficacy and more catastrophizing, which was moderately correlated with alexithymia and substantially correlated with affective pain and physical impairment [16]. This study is a cross-sectional study that sought to investigate the status of pain and its correlation with daily interference in childbearing-age women with MFPP through kinesiophobia, self-efficacy and pain catastrophizing, as well as to identify areas for future investigation and intervention based on the above findings. Pain intensity, daily interference, kinesiophobia (somatic focus and activity avoidance), self-efficacy, and pain catastrophizing (rumination, helplessness, and magnification) and their possible correlation were explored. Second, the potential involvement of pain catastrophizing and kinesiophobia in mediating the relationship between pain and daily interference was examined. Lastly, the possible independent or combined mediation role of self-efficacy in this population on the association between pain and the other outcomes of interest was investigated.

## Methods

### Study design

This was a hospital-based cross-sectional study conducted from 15 Nov 2022 to 10 Nov 2023. The Institutional Review Board of the primary hospital reviewed and approved this study on 02 Nov 2022 (No. I-22PJ633). Written informed consent was obtained before participants joined this study.

### Participants and settings

Childbearing-age women with clinically diagnosed MFPP (i.e., patients who self-reported moderate to severe pelvic pain that persisted for at least six months and recognition of pain trigger points on palpation assessment) attending partner hospitals to receive therapy were invited to participate in this study. A physician with more than ten years of experience in the management of pelvic floor dysfunction in women screened potential participants for eligibility. Inclusion criteria were female of reproductive age (18 to 49 years) who had ever had sex, non-menopause, and who had given informed consent. Pregnant women or participants with other pain-causing diseases (such as endometriosis, vaginal bleeding, and malignant tumors) were excluded from the study.

To guarantee the representativeness of the research findings, fourteen Grade A tertiary hospitals located in ten provinces of China (Liaoning in the Northeast, Beijing in the North, Henan and Hubei in the Central, Guangdong and Guangxi Zhuang Autonomous Region in the South, Qinghai in the Northwest, Yunnan in the Southwest, and Jiangsu and Shandong in the Eastern), which represent seven geographic and socioeconomic regions, were randomly selected as study regions.

### Data collection

During the research period, women aged 18–49 years with MFPP were consecutively recruited at the Department of Obstetrics and Gynecology or the Medical Rehabilitation Unit/Center of the fourteen partner hospitals. After ensuring patients understood the interview’s purpose, content and confidentiality, eligible patients were invited to participate in this study. Each participant self-reported a questionnaire on the online data management platform embedded in the WeChat application. The research team designed and verified the structured questionnaire, including demographic information, the Brief Pain Inventory, the Tampa Scale of Kinesiophobia, the Pain Self-Efficacy Questionnaire, and the Pain Catastrophizing Scale. For participants who were illiterate or had poor vision, the researchers verbally presented each item and option and verified that the questionnaires were filled out. As a result, all the data in this study is available.

### Measures

#### Socio-demographics

Characteristic variables and the potential risk factors for daily interference are as follows: (1) general characteristics included birth year, height (cm), weight (kg), occupation, education, marital status, monthly income (CNY); (3) history of pelvic surgery (yes/no) and childbirth (yes/no); (3) disease status included constipation(yes/no) and “currently diagnosed with gynecological disease” (yes/no); (4) MFPP treatment-related variables included “whether used over-the-counter medication for pain relief” (yes/no) and “previous pain treatment” (yes/no). In addition, body mass index (BMI) was calculated from height and weight, which were divided into four classes by tertile: <24.0 kg/m^2^ (underweight), 18.5–23.9 kg/m^2^ (normal), 24.0–27.9 kg/m^2^ (overweight), and ≥28.0 kg/m^2^ (obese).

#### The Brief Pain Inventory

The Brief pain inventory (BPI) is used to assess the pain severity over the last 24 hours and the impact of pain on daily interference [17]. The domain of pain severity assess pain at its “worst”, “least”, “average”, and “current status”, which is rated on an 11-point scale from 0 (no pain) to 10 (unbearable pain). The daily interference domain of BPI assesses how much pain has bothered physical and affective activities (i.e., general activity, walking, work, mood, enjoyment of life, relations with others, and sleep), which is also rated on an 11-point scale from 0 (not at all) to 10 (completely interferes) and then summed for a mean score. Both domains are recommended to be measured in all chronic pain research, and Cronbach’s alpha for the 4 and 7 items was 0.862 and 0.951, respectively.

#### The Tampa Scale of Kinesiophobia

Kinesiophobia, the level of fear about movement causing pain and injury was assessed using the 11-item version of the Tampa Scale of Kinesiophobia (TSK-11) [18]. Each scored item is rated on a 4-point scale ranging from 1 “strongly disagree” to 4 “strongly agree” (score range 11–44), and higher total scores indicate elevated levels of physical activity-related fear. The TSK-11 consisting of two subscales—“Somatic Focus” (TSK-SF; belief in underlying and serious medical problems) and “Activity Avoidance” (TSK-AA; belief that activity may result in (re)injury or increased pain), which has been shown to have good reliability and validity (Cronbach’s alpha for the TSK, TSK-SF, and TSK-AA in this study was 0.913, 0.866, and 0.888, respectively) [18].

#### The Pain Self-Efficacy Questionnaire

Pain-related self-efficacy, one’s confidence in performing a behavior despite experiencing pain, was assessed using the Pain Self-Efficacy Questionnaire (PSEQ) [19]. The PSEQ consists of 10 questions, with each item rated on a 7-point scale from 0 (not at all confident) to 6 (completely sure). The PSEQ has demonstrated good psychometric properties in individuals with pain, and Cronbach’s alpha in the current study was 0.964.

#### The Pain Catastrophizing Scale

Pain catastrophizing, the exaggerated or negative thoughts related to actual or anticipated pain (pain catastrophizing), was evaluated using the Pain Catastrophizing Scale (PCS) [20]. The PCS is a 13-item scale with three subscales: “Helplessness” (PCS-H; sense of helplessness to alleviate pain), “Magnification” (PCS-M; worries that something serious may happen), and “Rumination” (PCS-R; continually dwelling on the pain). Each scored item is rated on a scale of 0 (not at all) to 4 (all of the time), with higher scores indicating worse. A total PCS score of 30 is defined as “extreme catastrophizing”, which means positive for pain catastrophization. This scale has been validated for people with chronic pain, and Cronbach’s alpha for the PCS, PCS-H, PCS-M, and PCS-R in the present study was 0.963, 0.933, 0.857, and 0.914, respectively.

### Statistical analysis

Given the high consistency within each scale used in this study, the Item Parceling Strategy was employed, representing the dimension estimation with the mean value of items in each scale [21]. Since there were only eight variables in the final model: TSK-AA, TSK-SF, self-efficacy, PCS-rumination, PCS-magnification, PCS-helplessness, pain intensity, and daily interference, the minimum sample size for this study was 8 × 10 = 80, based on requirements of at least ten samples per variable. Furthermore, the recommended sample size for Structural Equation Modeling (SEM) is 200 [22], and this study ultimately enrolled 202 participants. Statistical analysis was conducted in SPSS 28.0, and two-sided with *P* < 0.05 was considered statistically significant.

Descriptive analysis and Pearson correlation were performed to examine the participants’ characteristics and variable associations. Means (*SD*), medians (*IQR*), or frequencies (percentage) were presented for the corresponding distribution of data. Linear regression was used to examine the factors associated with at least one favorable outcomes of the research (i.e., pain intensity, kinesiophobia, self-efficacy, pain catastrophizing, and daily interference) in reproductive-age women with MFPP, with the significant variables (*P* < 0.05) from the univariate analysis tested in the proposed mediating model. Only covariate that were significantly related to at least one variable of interest were included in the final serial mediating model.

Given the adequate sample size and approximately normal distributed data, AMOS 23.0 using a full information maximum likelihood estimator was employed to investigate the association between pain and daily interference and whether this is serial mediated by kinesiophobia, self-efficacy, and pain catastrophizing. The final estimated model was established using a goodness-of-fit criterion between the sample data and the hypothesis framework. The following indicators were used to assess the fitness: (1) Chi-square value to degrees of freedom (CMIN/df); (2) goodness of fit index (GFI); (3) root mean squared error of approximation (RMSEA); (4) Tucker-Lewis Index (TLI); (5) Comparative Fit Index (CFI). The CMIN/df with a value between 1 and 3, GFI, TLI, and CFI with a value over 0.9, and RMSEA with a value below 0.8 indicate a good fit.

Hayes’ PROCESS macro program (Model 6) based on OLS regression was used for testing the proposed serial mediation model. The mean value across items was used to present the estimations, the 95% bias-corrected confidence intervals (*CIs*) using 5000 bootstrapped samples, and adjustments were made iteratively to background characteristics such as age, BMI, monthly income, and reproductive history. If the *95% CI* does not include zero, then the direct and indirect effects are considered significant according to the above guidelines [23].

## Results

### Characteristics of participants

A total of 202 verified childbearing-age respondents with MFPP were included in this analysis. The mean age of the participants was 35.76 ± 6.51 years (22–49), and BMI was 22.10 ± 3.08 kg/m^2^. As shown in Table 1, most of the participants lived in urban (83.66%) and were married (89.11%); 106 participants (52.48%) had at least a college degree. 85 respondents (42.08%) had a monthly income less than CNY 5000, only 29 (14.36%) had not given birth, and 125 (61.88%) had a history of pelvic surgery. In addition, around half of the participants were currently diagnosed with gynecological disease (52.48%).

**Table 1:** Characteristic of participants (n = 202).

Variables	Total, n(%)
Mean age, yr (SD)	35.76 (6.51)
Age (years), %	
22–29	39 (19.31)
30–39	106 (52.48)
40–49	57 (28.22)
Mean BMI, kg/m^2^ (SD)	22.10 (3.08)
BMI, %	
Underweight (<18.5 kg/m^2^)	23 (11.39)
Normal (18.5–23.9 kg/m^2^)	131 (64.85)
Overweight (24–27.9 kg/m^2^)	37 (18.32)
Obese (≥28 kg/m^2^)	11 (5.45)
Location, %	
Urban	169 (83.66)
Rural	33 (16.34)
Occupation, %	
Intellectual labor	90 (44.55)
Manual labor	75 (37.13)
Retired/unemployed/other	37 (18.32)
Education, %	
Primary school or lower level	15 (7.43)
Middle or high school	81 (40.10)
College or higher level	106 (52.48)
Currently married, %	
No	22 (10.89)
Yes	180 (89.11)
Monthly income (CNY), %	
≤ 5000	85 (42.08)
>5000	67 (33.17)
>10000	26 (12.87)
>15000	14 (6.93)
>20000	10 (4.95)
Previous pelvic surgery, %	
No	77 (38.12)
Yes	125 (61.88)
Childbirth, %	
No	29 (14.36)
Yes	173 (85.64)
Currently diagnosed with gynecological disease, %	
No	96 (47.52)
Yes	106 (52.48)

#### Status of pain and the prevalence of pain catastrophization

In this study, only 9 (4.46%) reported using over-the-counter medication for pain relief, and almost half (46.04%) had been treated for MFPP. 95 (47.03%) participants reported moderate pain, and 83 (41.09%) were categorized as “extreme catastrophizers”. Participants reported the most daily interference with mood (mean score of 4.96) and the least with walking (mean score of 3.72) (Table 2).

**Table 2:** Pain status of participants (*n* = 202).

Pain status	Total, *n%*
Used over-the-counter medication for pain relief, %	
No	193 (95.54)
Yes	9 (4.46)
Previous pain treatment, %	
No	109 (53.96)
Yes	93 (46.04)
Pain intensity over the last 24 hours (0–10), mean (SD)	
Worst pain	5.08 (2.11)
Least pain	3.39 (2.22)
Average pain	4.30 (1.83)
Current pain	4.09 (2.23)
Level of the mean pain intensity, %	
No pain (0)	4 (1.98)
Mild (1–3)	86 (42.57)
Moderate (4–6)	95 (47.03)
Severe (7–10)	17 (8.42)
Kinesiophobia, mean (SD)	
TSK-SF (5–20)	14.52 (2.69)
TSK-AA (6–24)	16.58 (3.02)
Positive for pain catastrophization, %	
No	119 (58.91)
Yes	83 (41.09)
Pain catastrophizing, mean (SD)	
PCS-Helplessness (0–24)	11.11 (6.61)
PCS-Magnification (0–12)	5.60 (3.43)
PCS-Rumination (0–16)	8.78 (4.61)
Daily interference (0–10), mean (SD)	
General activity	4.69 (2.75)
Walking	3.72 (2.85)
Work	4.22 (3.03)
Mood	4.96 (2.80)
Enjoyment of life	4.43 (3.01)
Relations with others	4.07 (2.97)
Sleep	3.96 (2.88)

### Descriptive statistics and Pearson correlations of pain, kinesiophobia, self-efficacy, pain catastrophization, and daily interference

The mean pain was 4.21 ± 1.77 (scores), the mean kinesiophobia was 31.11 ± 5.19 (scores), the mean pain-related self-efficacy was 35.52 ± 16.93 (scores), the mean pain catastrophizing was 25.50 ± 13.92 (scores), and the mean daily interference was 4.22 ± 2.54 (scores) (Table 3). Based on the results of the Pearson correlations, participants perceived pain significantly correlated with kinesiophobia (*r* = 0.332, *P* < 0.001), pain catastrophizing (*r* = 0.432, *P* < 0.001) and daily interference (*r* = 0.586, *P* < 0.001), and correlated negatively with self-efficacy (*r* = -0.087, *P >* 0.05). The participants who reported a high level of kinesiophobia indicated weaker beliefs in self-efficacy (*r* = -0.539, *P* < 0.001) and more serious pain catastrophizing (*r* = 0.694, *P* < 0.001) and daily interference (*r* = 0.440, *P*<0.001). Pain-related self-efficacy correlated negatively with pain catastrophizing (*r* = -0.488, *P* < 0.001) and daily interference (*r* = -0.304, *P* < 0.001). The participants who reported a high level of pain catastrophizing indicated more serious daily interference (*r* = 0.620, *P* < 0.001).

**Table 3:** Descriptive statistics and Pearson correlations of pain-related variables.

Variables	M	SD	Min	Max	1	2	3	4	5
1. Pain intensity	4.21	1.77	0	10	1.000				
2. Kinesiophobia	31.11	5.19	11	44	0.332***	1.000			
3. Self-efficacy	35.52	16.93	0	60	-0.087	-0.539***	1.000		
4. Pain catastrophizing	25.50	13.92	0	52	0.432***	0.694***	-0.488***	1.000	
5. Daily interference	4.22	2.54	0	10	0.586***	0.440***	-0.304***	0.620***	1.000

M mean, Min minimum, Max maximum, SD standard deviation

***Signifcant correlation, *P* value < 0.001

### Measurement model

**Fig 1:**
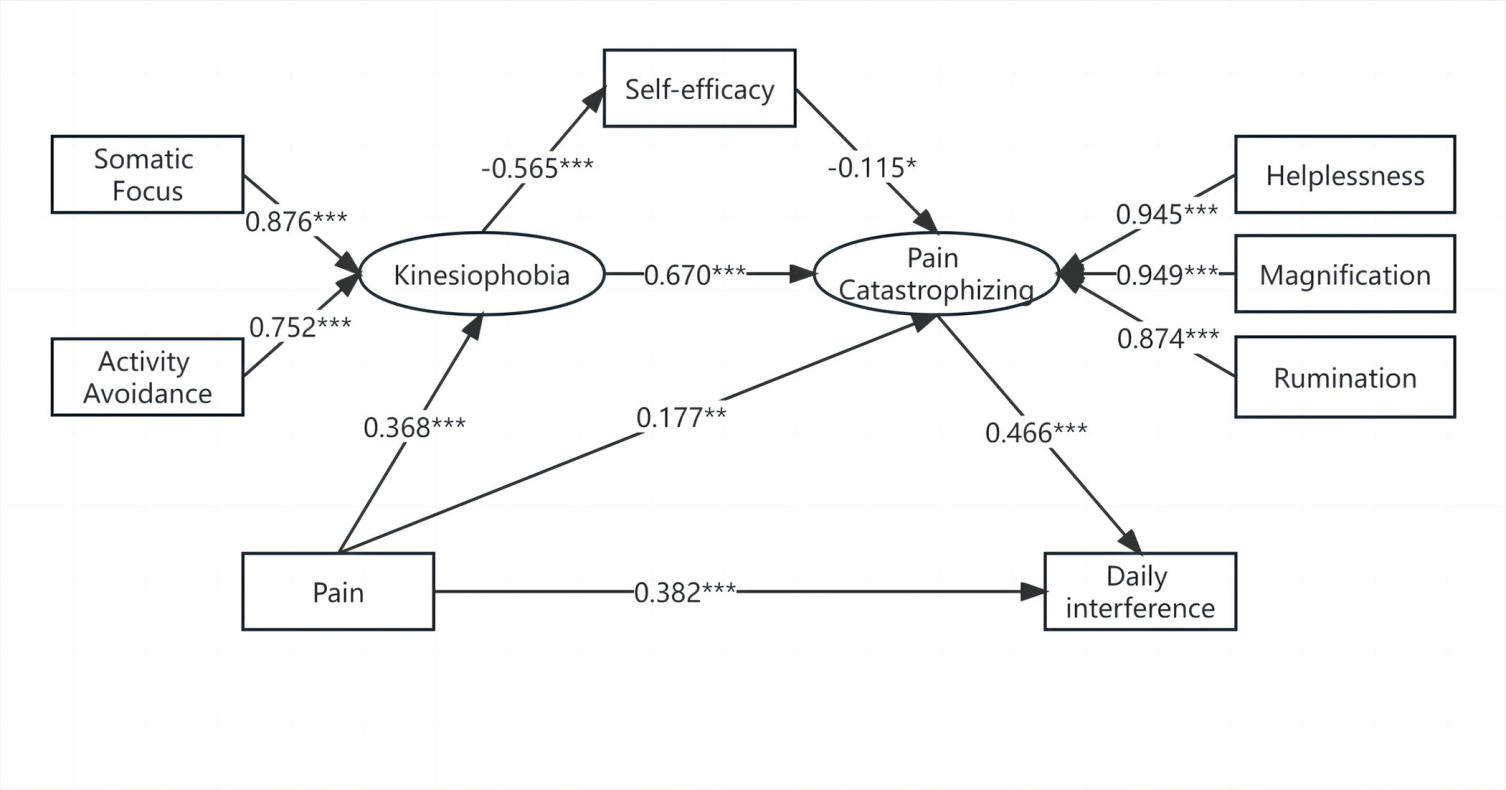
The serial mediation hypothesis with standardized estimates. Notes: *P<0.05; **P<0.01; ***P<0.001.

After removing the non-significant paths, the standardized fit indices indicated that the model was appropriate (see Fig 1): the CMIN/df was 1.995, GFI was 0.986, RMSEA was 0.070, TLI was 0.975, and the CFI was 0.986. For the factor loading of two subscales in TSK, “somatic focus” was 0.876 and “activity avoidance” was 0.752. For the factor loading of each subscale in PCS, helplessness was 0.945, magnification was 0.949, and rumination was 0.874.

### Covariates

According to bivariate correlation analysis, there were eight factors significantly related to at least one main variables of the research, including age, BMI, location, occupation, monthly income, childbirth, currently diagnosed with gynecological disease, and previous pain treatment (S1 Table).

### Test of serial mediation hypothesis

A mediation analysis was conducted using Model 6 in Hayes’ PROCESS to identify the possible serial mediating roles of kinesiophobia, self-efficacy, and pain catastrophizing in the association between pain and daily interference. First, we tested correlations between the variables in our proposed model and the potential covariates of age, BMI, location, occupation, monthly income, childbirth, currently diagnosed with gynecological disease, and previous pain treatment. As shown in Table 4, the first control variable, age,was significantly related to kinesiophobia (*β* = -0.112, *P* < 0.05) and self-efficacy (*β* = 0.497, *P* < 0.01). Income was also significantly related to kinesiophobia (*β* = -1.056, *P* < 0.001) and self-efficacy (*β* = 2.533, *P* < 0.01). Currently diagnosed with gynecological disease was associated with higher pain catastrophizing (*β* = 2.860, *P* < 0.05), and previous pain treatment was associated more serious daily interference (*β* = 0.590, *P* < 0.05), so them were incorporated into the research model as controls.

**Table 4:** Regression coefficients of serial mediation model.

Variables	Kinesiophobia		Self-efficacy		Pain catastrophizing		Daily interference	
	*β*	*95% CI*	*β*	*95% CI*	*β*	*95% CI*	*β*	*95% CI*
Pain intensity	0.887***	0.508, 1.265	0.678	-0.488, 1.844	1.792***	1.005, 2.580	0.571***	0.412, 0.729
Kinesiophobia			-1.652***	-2.064, -1.240	1.310***	0.992, 1.629	-0.019	-0.090, 0.051
Self-efficacy					-0.171***	-0.266, -0.076	-0.013	-0.032, 0.006
Pain catastrophizing							0.072***	0.044, 0.099
Age	-0.112*	-0.217, -0.007	0.497**	0.187, 0.808	-0.015	-0.229, 0.199	-0.010	-0.051, 0.031
Income	-1.056***	-1.639, -0.472	2.533**	0.771, 4.294	0.298	-0.911, 1.508	0.023	-0.208, 0.255
Gynecological diseases	0.351	-1.022, 1.723	1.15	-2.866, 5.167	2.860*	0.154, 5.565	0.311	-0.213, 0.836
Previous pain treatment	1.568	0.220, 2.916	-0.683	-4.676, 3.311	2.356	-0.332, 5.045	0.590*	0.071, 1.109
	*R*^*2*^ = 0.446		*R*^*2*^ = 0.599		*R*^*2*^ = 0.757		*R*^*2*^ = 0.728	
	*F*(196.000) = 5.000		*F*(195.000) = 6.000		*F*(194.000) = 7.000		*F*(193.000) = 8.000	

Note: Only covariates that were significantly related to at least one variable of interest in the serial mediating model were included in the table.

*Signifcant correlation, *P* value < 0.05.

**Signifcant correlation, *P* value < 0.01.

***Signifcant correlation, *P* value < 0.001.

In support of our hypothesis, pain had a positive direct link to kinesiophobia (*β* = 0.887, *P* < 0.001), pain catastrophizing (*β* = 1.792, *P* < 0.001), and daily interference (*β* = 0.571, *P* < 0.001). Kinesiophobia had a direct negative link to self-efficacy (*β* = -1.652, *P* < 0.001), and a positive direct link to pain catastrophizing (*β* = 1.310, *P* < 0.001). Lastly, as predicted, self-efficacy had a negative direct link to pain catastrophizing (*β* = -0.171, *P* < 0.001), and pain catastrophizing positively affected daily interference (*β* = 0.072, *P* < 0.001). However, there were no significant direct correlations between pain and self-efficacy, between kinesiophobia and daily interference, and between self-efficacy and daily interference (see Table 4).

**Fig 2:**
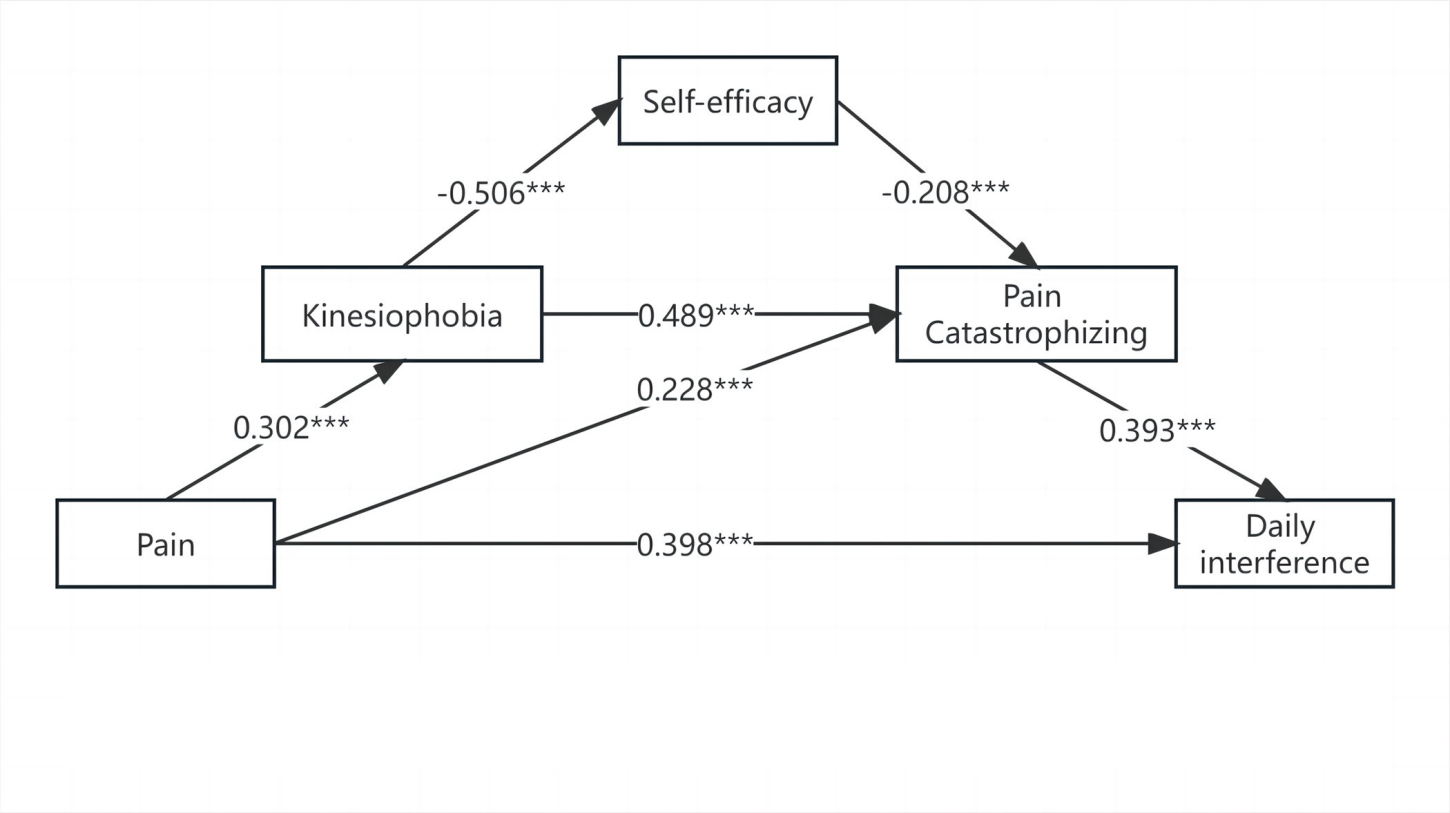
The final serial mediation model with standardized estimates. Notes: ***P<0.001; Age, monthly income, currently diagnosed with gynecological disease, and previous pain treatment were entered into the model as control variables.

Consistent with the hypothesis, our four-stage chain of mediation from pain to daily interference via higher kinesiophobia, lower self-efficacy, and higher pain catastrophizing in serial was significant (*P* < 0.05). Kinesiophobia and pain catastrophizing significantly mediated the relations between pain and daily interference. Pain catastrophizing also direct mediated the relationship between pain and daily interference. The remaining paths were not significant (See Table 5). The mean indirect (unstandardized) effect was 0.013, the standard error was 0.006, and the 95% confidence interval for the mean indirect effect was [0.003, 0.027]. In addition, the variables in the final regression analysis accounted for nearly three-quarters (*R*^*2*^  =  0.728) of the variance in daily interference (see Table 4 and Fig 2).

**Table 5:** Direct, indirect, and total effects.

Variables	*B*	*Boot SE*	*95%CI*
Pain-Daily interference	0.575***	0.081	0.415, 0.735
Indirect effect			
Pain-Kinesiophobia-Daily interference	-0.012	0.024	-0.061, 0.037
Pain-Self-efficacy-Daily interference	-0.008	0.011	-0.036, 0.006
Pain-Catastrophizing-Daily interference	0.088	0.028	0.038, 0.148
Pain-Kinesiophobia-Self-efficacy-Daily interference	0.014	0.013	-0.008, 0.044
Pain-Kinesiophobia-Catastrophizing-Daily interference	0.057	0.019	0.024, 0.098
Pain-Self-efficacy-Catastrophizing-Daily interference	-0.007	0.006	-0.021, 0.002
Pain-Kinesiophobia-Self-efficacy-Catastrophizing-Daily interference	0.013	0.006	0.003, 0.027
Total indirect effect	0.145	0.037	0.074, 0.219
Total effect (direct+indirect)	0.783***	0.083	0.620, 0.946

Note: Bootstrap samples for the bias-corrected interval is 5000; *B* regression coefficient; *Std*.*Error*, Standard error; *95% CI*, 95% confidence Interval; ***Signifcant correlation, *P* value < 0.001.

## Discussion

This study provided evidence for one possible explanation for the link between pain and daily interference, namely serial mediation via kinesiophobia, self-efficacy and, in turn, coping with pain catastrophizing among MFPP patients of childbearing age. As the findings show, pain could directly and significantly affect daily interference initially—secondly, pain catastrophizing partially mediated pain and daily interference. Thirdly, kinesiophobia and pain catastrophizing have a chain mediating effect. Lastly, pain also had an indirect impact on daily interference through the four-stage serial mediating of kinesiophobia, self-efficacy and catastrophizing.

### Direct influence of pain on daily interference

The study results showed that pain can directly affect daily interference in participants; they may have greater complaints of interference with their daily activities as the pain intensity increases. In terms of MFPP, it is usually related to gynecological, urological, and colorectal pain syndromes, and it can also manifest as visceral pain and muscular stretching discomfort in the bodily structures surrounding the pelvis [1]. Physical activities and routine work will unavoidably be impacted by the pain as it worsens, regardless of whether the person seeks medical attention at the hospital or adopts pain relief measures like sitting or lying still. Furthermore, it has been well-documented that chronic pain is significantly correlated with depressive symptoms and a sense of dissatisfaction with life [4]. A review has demonstrated that both pain and mood can interfere with sleep, such as being in too much pain or stressed to fall asleep or being awakened in the middle of the night [24]. Notably, sexual intercourse pain is another common symptom of MFPP in reproductive-age women; this discomfort is likely to compromise intimacy. Social distancing and depressive states following a period of chronic pain also have an impact on other social interactions and relationships with friends and family [25]. The findings imply that prioritizing the implementation of effective pain alleviation strategies is crucial to minimizing the daily disruptions induced by MFPP.

### Mediation effect of pain catastrophizing

We also investigated the underlying mechanisms of pain and daily interference. In line with the previous researches [6, 26, 27], this study findings reveal that pain catastrophizing showed strong and consistently significant influential role in the association between pain and daily interference, while kinesiophobia and self-efficacy have no independently mediating effect.

40.52% of MFPP patients had been categorized as “extreme catastrophizing” in our study, which was similar to the results of previous studies conducted among women with endometriosis in 2022 (40.74%), and women with chronic pelvic pain in 2015 (44.02%) [9, 28]. Despite the potential bidirectional link between pain and pain catastrophizing, a review of the basic neuroscience processes of pain suggests the stronger effect is pain predicting pain catastrophizing in clinical diagnostic pain conditions that were different from “psychogenic pain”, the direction tested in and supported by the current study [29]. Furthermore, Gellatly and Beck demonstrated that catastrophizing thinking might be a negative cognitive processing that contributes to a wide range of health and mental health conditions [8]. The correlation between pain catastrophizing and daily interference has been well documented [6, 8, 9, 27]. Extreme catastrophizers were identified in the sample population as being associated with strong interfered with many daily activities, even with the same level of pain and the same sense of failure to cure MFPP.

Moreover, these findings supported the functioning performance of pain catastrophizing (i.e., helplessness, magnification, and rumination) in the study participants, which shed light on a potential explanation for how pain catastrophizing affected everyday interference. Initially, chronic pain may induce a feeling of helplessness since one can only restrict one’s movements to alleviate the frequency and severity of the pain. In addition, due to the challenging nature of treating persistent pain, which is frequently misdiagnosed or overlooked in clinical settings, patients may develop concerns regarding the potential severity of the condition. Furthermore, MFPP patients also find it frustrating to put their discomfort out of their minds and concentrate on their jobs or other regular activities. As a result, people with chronic pain often find themselves caught in a vicious loop of avoidance or being hypervigilant about any daily task that could cause them pain, which makes their situation worse.

Among chronic pain patients, recovering from kinesiophobia and increased pain self-efficacy are associated with improvements in catastrophizing, physical functioning, and disability [7, 13, 30]. In this study, however, although playing a significant role in serial mediation after controlling for demographic factors, kinesiophobia and self-efficacy did not contribute significantly as a single mediator between pain and daily interference in the model. The lack of significance in the independent pathway through kinesiophobia or self-efficacy indicates that the existence of catastrophizing is necessary for both of which to help clarify the relationship between pain and pain interference. Similar consensus was reached in reviews that examined the psychological functioning of individuals with chronic pain, which demonstrated that elements of the FAM (especially for pain catastrophizing) appeared to be intrinsically linked to the experience of chronic pain, whereas self-efficacy did not appear to be [27, 31]. There is also empirical evidence linking pain catastrophizing to heightened activity in brain regions responsible for pain anticipation, emotion regulation, and physical motivations [32, 33]; improved kinesiophobia and self-efficacy, however, were only observed accompanying a reduction in catastrophic thinking among patients with chronic whiplash associated disorders after receiving target therapies [34].

### Serial mediation effect of kinesiophobia, self-efficacy and pain catastrophizing

Consistent with our primary hypothesis, the significant serial mediational pathway suggests that reproductive-age women with MFPP may respond to kinesiophobia and self-efficacy following the pain caused by the pelvic myofascial disorder to pain catastrophizing, which in turn puts them at risk for daily interference. Previous research has indicated that kinesiophobia is associated with pain intensity in people suffering from chronic pain, and high level of kinesiophobia links reduced self-efficacy among patients with non-specific low back pain [35, 36]. In addition, the reduction in self-efficacy can enhance the thinking of pain catastrophizing in patients with chronic pain [32]. As aforementioned, pain catastrophizing is positively related to daily interference [8, 9]. Patients who had a higher level of pain catastrophizing tended to have greater daily interference.

Our findings suggested that more attention should be paid to childbearing-age patients with severe interference with daily life, and pain target therapies aimed at relieving pain, decreasing pain catastrophizing and kinesiophobia, and strengthening self-efficacy should be adopted by medical personnel. To begin with, patients need to receive the clinically recommended therapies for MFPP, such as electrical stimulation and releasing techniques, to address the root cause of their daily interference [1]. Consequently, early identification of target patients who exhibit high levels of catastrophizing thinking and kinesiophobia, or low self-efficacy before prescribing any treatment may facilitate clinical decision-making. Finally, depending on their evaluation results, patients are encouraged to receive targeted and interdisciplinary interventions that have been identified as effective at improving function, mood, and pain interference among people with chronic pain [37, 38].

### Limitations and suggestions for future research

First, while patients were enrolled in this research from hospitals across seven geographical regions in China, generalizing the findings was limited since there were more recruiting centers in southeast China and fewer in northwest China (particularly in minority autonomous regions). It is suggested that future research consider regional equalization of patient recruitment to reduce selection bias. Furthermore, nearly half of the patients had undergone unsuccessful prior treatments for their conditions, which may indicate a higher level of pain catastrophization among the study participants. Future studies could screen out newly diagnosed patients at the enrollment stage and compare the findings to the current study’s conclusions to further perfect the theoretical model and better understand the impact of pain catastrophizing on daily interference in reproductive-age women with MFPP. Additionally, while our findings support a partial mediation, the presence of other potential mediators or moderators that were not considered in this study should be evaluated and further examined. Finally, due to the cross-sectional design, the study could not confirm cause-and-effect correlations between variables, and it was also impossible to figure out what role other variables, like resilience or stress, played in the pathway from pain to daily interference. Based on this, future research could consider expanding long-term longitudinal analysis to validate the theoretical model and demonstrate causal relationships. Also, it would be noteworthy to investigate the effects of future interventions aimed to strengthen self-efficacy in reproductive-age women with MFPP and manage kinesiophobia and pain catastrophizing.

### Relevance for clinical practice

Measurements of pain catastrophizing, kinesiophobia, and self-efficacy should be incorporated into pain assessment for reproductive-age women with MFPP in the Medical Rehabilitation Units/Centers, as these pain-related cognitive factors may be significant therapy objectives to drive the relationship between pain intensity and daily interference. Following this, psychological therapies or rehabilitation programs should incorporate strategies that decrease pain catastrophizing and kinesiophobia while boost self-efficacy, such as acceptance and commitment therapy, cognitive behavioral therapy and mindfulness-based intervention [37, 38]. As a result, reproductive-age women with MFPP may be able to alleviate their perception of pain, enhance their health-related quality of life, and embrace the opportunity to approach the stage of pregnancy preparation without concern.

## Conclusions

According to the study findings, the association between pain and daily interference among reproductive-age women with MFPP can be explained in four ways. Pain influences the interference of daily activities, not only through its direct effects and the simple mediation of catastrophizing, but also through the sequential mediation of kinesiophobia and pain catastrophizing, and the four-stage serial mediation of kinesiophobia, self-efficacy and pain catastrophizing. For future clinical applications, kinesiophobia and self-efficacy, particularly pain catastrophizing, could be crucial psychological therapy targets for reproductive-age women with MFPP seeking to alleviate their pain perception and improve quality of life.

## Supporting information

S1 Table Bivariate correlations for main study variables. (DOCX)

S1 Dataset The whole dataset for this study. (XLSX)

S1 Appendix Statistical methods. (DOCX)

## Abbreviations

MFPP: Myofascial pelvic pain
FAM: : the Fear-Avoidance Model
CNY: Chinese Yuan
BMI: Body Mass Index
BPI: the Brief pain inventory
TSK-11: the 11-item version of the Tampa scale of kinesiophobia
TSK-SF: the subscale of “Somatic Focus” in TSK-11
TSK-AA: the subscale of “Activity Avoidance” in TSK-11
PSEQ: the Pain Self-Efficacy Questionnaire
PCS: the Pain Catastrophizing Scale
PCS-H: the subscale of “Helplessness” in PCS
PCS-M: the subscale of “Magnification” in PCS
PCS-R: the subscale of “Rumination” in PCS
SEM: Structural Equation Modeling
SD: Standard Deviation
IQR: Interquartile range
P: Probability
95% CI: 95% Confidence Interval
CMIN/df: Chi-square value to degrees of freedom
GFI: goodness of fit index
RMSEA: root mean squared error of approximation
TLI: Tucker-Lewis Index
CFI: Comparative Fit Index
