# Supplementary material for: Pain and daily interference among reproductive-age women with myofascial pelvic pain: Serial mediation roles of kinesiophobia, self-efficacy and pain catastrophizing
Source: PLoS One. 2024 May 13;19(5):e0301095. doi: 10.1371/journal.pone.0301095 (PMC11090321; doi:10.1371/journal.pone.0301095)
Supplement: S1 Table — (DOCX) [file pone.0301095.s001.docx]

S1 Table

| **S1 Table Bivariate correlations for main study variables** | | | | | | | | | | |
| --- | --- | --- | --- | --- | --- | --- | --- | --- | --- | --- |
| **Variables** | **Pain intensity** | | **Kinesiophobia** | | **Self-efficacy** | | **Pain catastrophizing** | | **Daily interference** | |
|  | ***β (95% CI)*** | ***P*** | ***β (95% CI)*** | ***P*** | ***β (95% CI)*** | ***P*** | ***β (95% CI)*** | ***P*** | ***β (95% CI)*** | ***P*** |
| **Age** | 0.029 (-0.009, 0.066) | 0.133 | -0.051 (-0.162, 0.060) | 0.363 | 0.578 (0.224, 0.931） | 0.001** | -0.062 (-0.360, 0.236) | 0.680 | 0.005 (-0.049, 0.059) | 0.854 |
| **BMI** | 0.102 (0.023, 0.181) | 0.011* | 0.176 (-0.058, 0.410) | 0.139 | -0.203 (-0.970, 0.563) | 0.601 | 0.445 (-0.183, 1.072) | 0.164 | 0.133 (0.020, 0.247) | 0.021* |
| **Location** | | | | | | | | | | |
| Urban | Reference |  | Reference |  | Reference |  | Reference |  | Reference |  |
| Rural | 0.760 (0.103, 1.417) | 0.024* | 1.862 (-0.074, 3.798) | 0.059 | -7.400 (-13.687, -1.114) | 0.021* | 5.632 (0.453, 10.811) | 0.033* | 0.716 (-0.233, 1.665) | 0.138 |
| **Occupation** | | | | | | | | | | |
| Intellectual labor | Reference |  | Reference |  | Reference |  | Reference |  | Reference |  |
| Manual labor | 0.126 (-0.422, 0.673) | 0.652 | -0.038 (-1.633, 1.558) | 0.963 | -2.633 (-7.810, 2.543) | 0.317 | 0.127 (-4.173, 4.426) | 0.954 | -0.111 (-0.896, 0.675) | 0.781 |
| Retired/ unemployed/ other | 0.028 (-0.656, 0.712) | 0.936 | 1.702 (-0.290, 3.695) | 0.094 | -7.630 (-14.096, -1.164) | 0.021* | 3.019 (-2.351, 8.389) | 0.269 | 0.101 (-0.880, 1.083) | 0.839 |
| **Education** | | | | | | | | | | |
| Primary school or lower level | Reference |  | Reference |  | Reference |  | Reference |  | Reference |  |
| Middle or high school | -0.591 (-1.573, 0.390) | 0.236 | -0.358 (-3.249, 2.533) | 0.807 | -1.775 (-11.200, 7.650) | 0.711 | 0.946 (-6.807, 8.698) | 0.810 | 0.522 (-0.886, 1.931) | 0.466 |
| College or higher level | -0.381 (-1.344, 0.582) | 0.436 | -0.154 (-2.992, 2.684) | 0.915 | -0.438 (-9.688, 8.811) | 0.926 | 0.103 (-7.505, 7.712) | 0.979 | 0.716 (-0.666, 2.099) | 0.308 |
| **Currenly married** | | | | | | | | | | |
| No | Reference |  | Reference |  | Reference |  | Reference |  | Reference |  |
| Yes | 0.239 (-0.550, 1.028) | 0.551 | -1.255 (-3.566, 1.056) | 0.286 | 5.639 (-1.880, 13.158) | 0.141 | -3.622 (-9.817, 2.574) | 0.250 | -0.573 (-1.702, 0.557) | 0.319 |
| **Monthly income (CNY)** | | | | | | | | | | |
| ≤ RMB 5000 | Reference |  | Reference |  | Reference |  | Reference |  | Reference |  |
| >RMB 5000 | -0.068 (-0.639, 0.503) | 0.814 | -0.026 (-1.655, 1.602) | 0.975 | 3.552 (-1.735, 8.839) | 0.187 | -0.493 (-4.945, -3.959) | 0.827 | -0.610 (-1.427, 0.207) | 0.142 |
| >RMB 10000 | -0.656 (-1.440, 0.127) | 0.100 | -2.309 (-4.542, -0.075) | 0.043* | 10.958 (3.705, 18.210) | 0.003** | -6.011 (-12.118, 0.096) | 0.054 | -0.978 (-2.099, 0.143) | 0.087 |
| >RMB 15000 | 0.117 (-0.891, 1.125) | 0.819 | -2.561 (-5.436, 0.314) | 0.080 | 13.112 (3.778, 22.446) | 0.006** | -4.879 (-12.739, 2.981) | 0.222 | -0.355 (-1.797, 1.087) | 0.628 |
| >RMB 20000 | -0.354 (-1.523, 0.814) | 0.550 | -5.147 (-8.479, -1.815) | 0.003** | 12.912 (2.093, 23.730) | 0.020* | -7.865 (-16.975, 1.246) | 0.099 | -0.682 (-2.353, 0.990) | 0.422 |
| **Childbirth** | | | | | | | | | | |
| No | Reference |  | Reference |  | Reference |  | Reference |  | Reference |  |
| Yes | -0.003 (-0.705, 0.699) | 0.993 | -2.087 (-4.126, -0.049) | 0.045* | 2.425 (-4.284, 9.133) | 0.477 | -4.087 (-9.580, 1.407) | 0.144 | -0.027 (-1.033, 0.979) | 0.958 |
| **Previous pelvic surgery** | | | | | | | | | | |
| No | Reference |  | Reference |  | Reference |  | Reference |  | Reference |  |
| Yes | 0.444 (-0.059, 0.947) | 0.083 | 0.617 (-0.868, 2.101) | 0.414 | 2.989 (-1.843, 7.820) | 0.224 | 2.445 (-1.528, 6.418) | 0.226 | 0.571 (-0.151, 1.293) | 0.120 |
| **Currently diagnosed with gynecological disease** | | | | | | | | | | |
| No | Reference |  | Reference |  | Reference |  | Reference |  | Reference |  |
| Yes | 0.391 (-0.099, 0.880) | 0.117 | 0.545 (-0.899, 1.989) | 0.458 | 2.151 (-2.555, 6.858) | 0.368 | 4.248 (0.416, 8.080) | 0.030* | 0.863 (0.167, 1.559) | 0.015* |
| **Used over-the-counter medication for pain relief** | | | | | | | | | | |
| No | Reference |  | Reference |  | Reference |  | Reference |  | Reference |  |
| Yes | 1.085 (-0.097, 2.268) | 0.072 | 1.747 (-1.745, 5.238) | 0.325 | -3.922 (-15.324, 7.480) | 0.498 | 4.477 (-4.888, 13.842) | 0.347 | 1.162 (-0.540, 2.864) | 0.180 |
| **Previous pain treatment** | | | | | | | | | | |
| No | Reference |  | Reference |  | Reference |  | Reference |  | Reference |  |
| Yes | 0.622 (0.136, 1.108) | 0.012* | 2.189 (0.773, 3.606) | 0.003** | -3.463 (-8.164, 1.237) | 0.148 | 7.343 (3.595, 11.091) | <0.001*** | 1.510 (0.835, 2.186) | <0.001** |
| *Signifcant correlation, P value<0.05. | | | | | | | | | | |
| **Signifcant correlation, P value<0.01. | | | | | | | | | | |
| ***Signifcant correlation, P value<0.001. | | | | | | | | | | |
