# Supplementary material for: Pain and daily interference among reproductive-age women with myofascial pelvic pain: Serial mediation roles of kinesiophobia, self-efficacy and pain catastrophizing
Source: PLoS One. 2024 May 13;19(5):e0301095. doi: 10.1371/journal.pone.0301095 (PMC11090321; doi:10.1371/journal.pone.0301095)
Supplement: S1 Appendix — (DOCX) [file pone.0301095.s003.docx]

**S1 Appendix**

Statistical methods

| **Study’s findings** | **Methods** | **The corresponding variable names** |
| --- | --- | --- |
| Table 1, Table 2 | SPSS 28.0; Descriptive analysis were performed to examine the participants’ characteristics. Means (*SD*), medians (*IQR*), or frequencies (percentage) were presented for the corresponding distribution of data. | age, age_3, bmi, bmi_4, location, occupation, education, marry, income, surgery, childbirth, gynecology, medication, treatment, pain_worst, pain_least, pain_average, pain_current, pain_mean4, TSK_SF, TSK_AA, catastrophization, PCS_H, PCS_M, PCS_R, Daily interference_activity, Daily interference_walking, Daily interference_work, Daily interference_mood, Daily interference_enjoyment, Daily interference_relations, Daily interference_sleep |
| Table 3 | SPSS 28.0; Pearson correlation was performed to examine the variable associations. | Pain intensity, Kinesiophobia, Self-efficacy, Pain catastrophizing, Daily interference |
| Fig.1 | AMOS 23.0 using a full information maximum likelihood estimator was employed to investigate the association between pain and daily interference and whether this is serial mediated by kinesiophobia, self-efficacy, and pain catastrophizing. The final estimated model was established using a goodness-of-fit criterion between the sample data and the hypothesis framework. The CMIN/df with a value between 1 and 3, GFI, TLI, and CFI with a value over 0.9, and RMSEA with a value below 0.8 indicate a good fit. | TSK_SF, TSK_AA, PCS_H, PCS_M, PCS_R, Self-efficacy, Pain intensity, Daily interference |
| S1 Table | SPSS 28.0; Linear regression was used to examine the factors associated with at least one favorable outcomes of the research (i.e., pain intensity, kinesiophobia, self-efficacy, pain catastrophizing, and daily interference) in reproductive-age women with MFPP, with the significant variables (P < 0.05) from the univariate analysis tested in the proposed mediating model. | Pain intensity, Kinesiophobia, Self-efficacy, Pain catastrophizing, Daily interference, age, bmi, location, occupation, education, marry, income, childbirth, surgery, gynecology, medication, treatment |
| Table 4, Table 5, Fig.2 | SPSS 28.0 and Hayes’ PROCESS macro program (Model 6) based on OLS regression was used for testing the proposed serial mediation model. The mean value across items was used to present the estimations, the 95% bias-corrected confidence intervals (CIs) using 5000 bootstrapped samples, and adjustments were made iteratively to background characteristics including age, BMI, location, occupation, monthly income, childbirth, currently diagnosed with gynecological disease, and previous pain treatment. If the 95% CI does not include zero, then the direct and indirect effects are considered significant according to the above guidelines | Pain intensity, Kinesiophobia, Self-efficacy, Pain catastrophizing, Daily interference, age, bmi, location, occupation, income, childbirth, gynecology, treatment |
